# Facial Expression Recognition with Geometric Scattering on 3D Point Clouds

**DOI:** 10.3390/s22218293

**Published:** 2022-10-29

**Authors:** Yi He, Keren Fu, Peng Cheng, Jianwei Zhang

**Affiliations:** 1 National Key Laboratory of Fundamental Science on Synthetic Vision, Chengdu 610065, China; 2 College of Computer Science, Sichuan University, Chengdu 610065, China; 3 School of Aeronautics and Astronautics, Sichuan University, Chengdu 610065, China

**Keywords:** 3D facial expression recognition, geometric scattering, point clouds

## Abstract

As one of the pioneering data representations, the point cloud has shown its straightforward capacity to depict fine geometry in many applications, including computer graphics, molecular structurology, modern sensing signal processing, and more. However, unlike computer graphs obtained with auxiliary regularization techniques or from syntheses, raw sensor/scanner (metric) data often contain natural random noise caused by multiple extrinsic factors, especially in the case of high-speed imaging scenarios. On the other hand, grid-like imaging techniques (e.g., RGB images or video frames) tend to entangle interesting aspects with environmental variations such as pose/illuminations with Euclidean sampling/processing pipelines. As one such typical problem, 3D Facial Expression Recognition (3D FER) has been developed into a new stage, with remaining difficulties involving the implementation of efficient feature abstraction methods for high dimensional observations and of stabilizing methods to obtain adequate robustness in cases of random exterior variations. In this paper, a localized and smoothed overlapping kernel is proposed to extract discriminative inherent geometric features. By association between the induced deformation stability and certain types of exterior perturbations through manifold scattering transform, we provide a novel framework that directly consumes point cloud coordinates for FER while requiring no predefined meshes or other features/signals. As a result, our compact framework achieves 78.33% accuracy on the Bosphorus dataset for expression recognition challenge and 77.55% on 3D-BUFE.

## 1. Introduction

Facial expression recognition (FER) is a prevalent artificial intelligence and machine perceptual research fields relating to the divergent evolution of data representation techniques. The most common branch is based on image/video signals, and focuses on learning to extract expression features from variables embedded in the regular (grid-like) space, allowing Euclidean models such as CNNs/SVMs/PCA to extract salient features from spatial/temporal correlations [1,2]. Methods combining 3D and 2D [3,4,5] commonly try to restore a geometrical representation from multi-modal sensing results, e.g., raster images and point cloud scans, and then apply the texture channel as the auxiliary salient information to overcome possible perturbations such as isometry in real 3D scenes. For instance, the rigid rotation augmentation scheme called Multi-View Stereo (MVS) is a representative member [6]; however, this branch requires exhausted representation interpolation, which limits its realistic application and incurs extra exterior noise. Furthermore, issues in real recognition scenarios such as illumination variations and occlusions restrain the generality and robustness of 2D-based methods, as the conspicuousness of an expression as the key inter-variance has the risk of being repressed by inter-subject variances and environmental noises. Meanwhile, the 2D representation inevitably entangles the above elements.On the other hand, 3D representations, including meshes/point clouds, sustain a more stable underlying geometrical structure against various types of interference, e.g., isometry, sampling frequency shifting, and other largely irrelevant environmental variables such as luminance disturbance in raster image sensing processes. To sum up, a proper representation for 3D FER should be invariant to unrelated variations caused by extrinsic elements while reserving local characterized deformations that often act in subtler magnitudes or higher frequencies. The ultimate target should be to classify such different manifolds ${\left\{M\right\}}_{j=1}^{N_{expression}}$, where class-specific local isometrics/diffeomorphism should be considered as the inherent source of salient features. Geometric deep learning (GDL) has been introduced in [7], extending classical CNN into the so-called non-Euclidean geometry setting and exploring the intrinsic information from observed data with a deep network structure. It has shown vitality in multiple disciplines, including [8,9,10] relating to 3D vision, to name only a few. This framework has profoundly proved itself in studying the underlying non-Euclidean geometry of observed data with geometric priors prescribed in advance. With this capability, good interpretability and efficiency concerning questions involving geometrically represented data can be reasonably expected. Because the 3D FER question involves complex underlying geometry, it is necessary to adapt raw data into a more regular form with redundant manipulations before applying traditional Euclidean deep learning methods. The intuitive approach of finding a direct approach to consuming raw 3D data with GDL turns out to be essential.

In this paper, we follow [11] by consuming pure 3D point cloud coordination as the only data while alternatively assuming expression samples to be geometric objects lying on characterized low-dimensional manifolds. To accomplish this idea, we apply the geometric wavelet scattering transform [12] in 3D FER in a novel way; our experimental results on the Bosphorus [13] and 3D-BUFE [14] datasets prove the high efficiency of this approach in terms of balancing expressiveness with stability against multiple random deformation or noise. By synchronously extracting a localized and interior spatial feature from raw point clouds with less pre-processing, our proposed approach maintains a relatively shallow and straightforward structure.

Our contributions can be summarized as follows:

- We propose a localized and inherent density descriptor to reserve the fine geometry of faces without requiring a predefined mesh structure.
- We introduce a manifold scattering transform to integrate such local features into a manifold to maintain a common coarse underlying geometry characterized by a few landmark points. This method shows robustness against exterior perturbations while reserving discrimination for FER.
- The proposed method is purely based on 3D point clouds/sets and does not use any meshes or textures. Compared to state-of-the-art (SOTA) solutions of the same type, our method shows improved accuracy by ∼8% when classifying the seven expressions on Bosphorus dataset.

The remainder of this paper is organized as follows. In Section 2, we list related works and trends in 3D FER. In Section 3, we describe our overall framework and detail our proposed methods, including the local density descriptor and manifold scattering transform. In Section 4, we summarize the datasets and corresponding evaluation protocol utilized in this work, provide the hyper-parameter tuning process, state the major results, and present a comparison with competing methods. Finally, in Section 5, we provide a discussion of the topic and conceivable future extensions.

## 2. Related Works

3D FER methods have succeeded in mining prominent geometrical details by SPD (Symmetric Positive Matrix) [15], conformal mapping [3], depth map [16], and recently with the prevalent statistical 3D Morphable Model (3DMM) [17] and point sets [11]. Among these, Refs. [1,2] can partially solve the above isometric issues, although the texture channel is required as a discriminative feature. The stereo matching-based method [18] has shown advantages in dealing with rigid transformation in identification scenarios. In the geometric learning branch, Ref. [15] relies on a predefined mesh structure, while [11] has been the first to adopt a geometric deep learning framework (i.e., PointNet [19]) with 3D FER. This inspires our motivation to explore the above approach in order to realize further improvements in performance. For 3D FER and other real scenario scan data, the non-uniform sampling condition and the complexity of local deformation caused by the mixture of expression and other exterior perturbations (e.g., pose variation, the subject’s characteristic biological shape) can increase the intrinsic dimension of the observed realizations. Embedding prefabricated features into such a Riemann manifold incurs extra risk of warping feature signals and failing to achieve non-aliasing asymptotic aspects. A common approach to deal with this problem relies on considering an approximately smooth and compact Riemann manifold as the underlying geometry of an expression in order to obtain differentiability, then embedding an exterior and preprocessed generic function to further enhance discrimination. Examples include SHOT [20], HoG descriptor [21], and curvature mean maps [22]. Furthermore, facial recognition has seen the implementation of similar methods [23,24]; an overall projection matrix was used to assemble sub-patterns, although this approach implicitly assumed the elimination of sampling or environmental noise. A brief summary of the above-mentioned works is provided in Table 1, showing the modality and representation methods and classifying the different models.

In a prior trial in 3D FER with GDL, Ref. [11] utilized PointNet to abstract multi-scale features from raw point clouds by stacking set abstraction layers. This can be considered as an increasing path of “receptive fields”, with the common disadvantage of a loss of finer local geometry in each hierarchical layer. An abstraction operation coarsely reservs principle components from the local region, and the corresponding drop-out layer helps to forbid over-fitting; however, it severely abates the expressiveness of the point cloud representation. More recent approaches [12,27] have provided an alternative solution based on defining a deep representation that is invariant to isometric transformations up to an induced small scale. These methods rely on the idea of constructing class-specified stability on high-dimensional representations. Specifically, Ref. [27] provided a framework for smoothing high dimensional representation into the general manifold using manifold heat interpolation [28] and a multi-scale wavelets filter bank network [29], thereby loosening the prior condition with respect to the compactness and smoothness of the manifold.

## 3. Methodology

**Figure 1:**
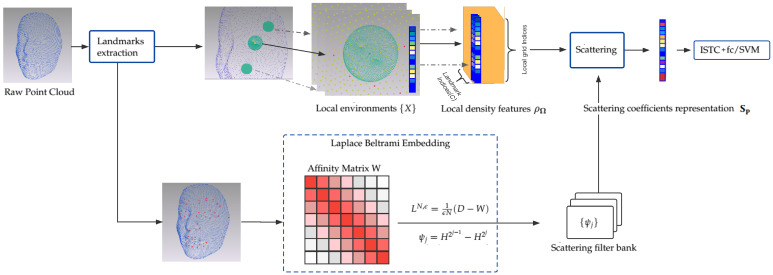
Overall framework for 3D FER.

The overall framework is illustrated in Figure 1. First, manual landmark points (22 for the Bosphorus dataset and 83 for the 3D-BUFE dataset) are obtained from raw point clouds. Our method then treats them as the starting point for two synchronous routes. In the first route (the upper route in Figure 1), we consider each landmark as the origin of a discretized Gaussian kernel with adaptive width, followed by a KNN search to determine its neighbors, the Euclidean radial distances of which inversely contribute to computing the smoothed local spatial representation. By and large, this function is one form of the classical kernel density estimation [30] scheme, which preserves the simplicity while regularizing the scattered position representation into a continuous form without losing resolution. Though this description is extrinsic in terms of sensitivity to local permutation of the indexing/order, the manifold scattering transform can help to regularize the underlying overall geometry of the expression manifold; see Section 3.1 for details of this spatial density descriptor.

The second route (the lower route in Figure 1) involves finding a common structure in order to identify the expression from the raw point cloud representation. This structure should devote itself to representing identity-unrelated parts. In 3D FER, a self-evident condition is that both expressions and other identity-unrelated behaviors affect mostly local regions, which can include stretching, local rotation, and other diffeomorphisms. In this intuition, we find that diagonalizing the affinity matrix of the landmarks set and then computing the corresponding diffusion structures (similar to graph network embedding solutions [31,32], which imitate a discrete approximation Laplacian Beltrami Operator (LBO) on a manifold) has a good chance of achieving this goal. Note that because the landmark set is quite small, the eigenvectors of a heat kernel *H* are easy to obtain and should be sufficient to represent the coarse geometry of an expressed face. Specifically, the raw point cloud face scan is treated as a small graph built from the landmarks set; in our example, 22/83 landmarks from a Bosphorus/3D-BUFE sample could be spectral decomposed into eigenvalues and eigenfunctions, with the *K* top components then truncated and fed to parameterize the scattering filter bank and the corresponding network; the details of implementing this parameterization can be found in Section 3.2.

Finally, by equipping prevalent classifiers, e.g., SVM/Neural Networks, certain components of the signal are selected to enhance the recognition accuracy. Specifically, we note that the high dimensional nature of scattering coefficients representation $S_{f}$ may lead to overfitting during training. Therefore, a sparse learning structure [33] is inserted to provide non-linearity and enhance the sparsity of features, which are then fed to the fully connected layer for classification. An improvement in accuracy can be observed afterward, and we compare the performance using the SVM with an RBF (radial basis function) kernel as the classifier. The details of these experiments are reported in Section 4.

### 3.1. Local Density Descriptor

At first, the raw point cloud’s high dimensionality tends to diminish the ability of Euclidean convolution or other deep learning methods that hold prior assumptions as to the signal’s properties, such as its smoothness and compactness. Moreover, unlike the body meshes utilized in [27], face scan samples have more complex local geometries and irregular overall variance distributions, which increases the probability of overfitting or gradient explosion in the training phase. On the other hand, an isometry-invariant local descriptor and mesh reconstruction scheme can block the development of a real-time-capable approach. In this case, we apply a lighter local feature extraction approach to describe the expression manifolds with the occurrence probability density of the local point clusters and aggregate the localities by computing the eigenvector of landmark points and constructing the corresponding semi-group diffusion heat maps to surmount the prevalent existence of sampling non-uniformity.

We suppose a face scan, denoted as $x_{i}$, within which we extract *C* landmark points, denoted as a landmark set ${\left\{p_{landmark_{c}}\right\}}_{c=1}^{C}$; we then embed this into a small graph G=(Ω,W), where Ω is the landmark point index set and W is a C×C symmetric matrix.

**Figure 2:**
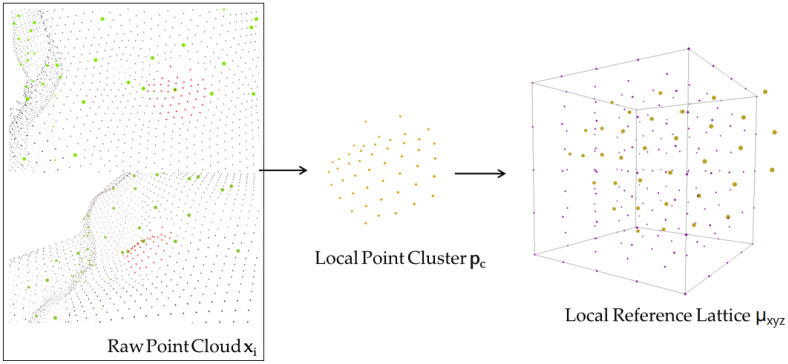
From left to right: a neighboring point cluster has been extracted from the raw point cloud (points in red), from the centroid of which a local reference lattice is constructed for further computation.

The following process of obtaining a local feature starts with the construction of local reference frames centered at each landmark point $p_{landmark_{c}}$, which can be seen as a local “atomic environment” χ (See Figure 2). In each χ, a small face patch around each landmark point shares a generic pattern relating to expressions across any subject. By aligning a kernel function based on the distribution of *N* observed points ${\left\{r_{n}\right\}}_{n=1}^{N}$ in each local reference environment χ, the resulting local probability density of observing a point at grid positions within each environment is a smoothed and discriminative representation/feature

(1) $${\hat{\rho }}_{\chi }\left(\mu \right)=\sum_{n=1}^{N}exp\left(-\frac{{\left(\mu -r_{n}\right)}^{2}}{2\sigma^{2}}\right)$$

which is a sum of Gaussian functions at the local regular reference lattice function μ. This kernel maps the Euclidean distance from scattered points ${\left\{r_{n}\right\}}_{n=1}^{N}$ to a probability distribution and slices each $x_{i}$ into C local receptive fields; by normalizing each density function according to the adjustment of σ, it eventually defines a global piece-wise density representation

(2) $${\hat{\rho }}_{\Omega }\left(\mu \right)=\left({\hat{\rho }}_{1},\cdots ,{\hat{\rho }}_{c},\cdots ,{\hat{\rho }}_{C}\right).$$

The above approach encodes a raw point cloud face into a more regular continuous probability density representation, with local fields being invariant to permutations of the input order and each characteristic vector holding a correspondent length, thereby enabling windowed operations. Moreover, the length of local point sets can be arbitrary, and non-uniform sampling affects the results as an additive bias to the signal on each grid point.

The isometry within each local area can be treated individually by adopting the coarse graph embedding induced by each sample’s sparse landmarks set, with the induced wavelet filters parameterized to the corresponding direction decided thereby.

**Figure 3:**
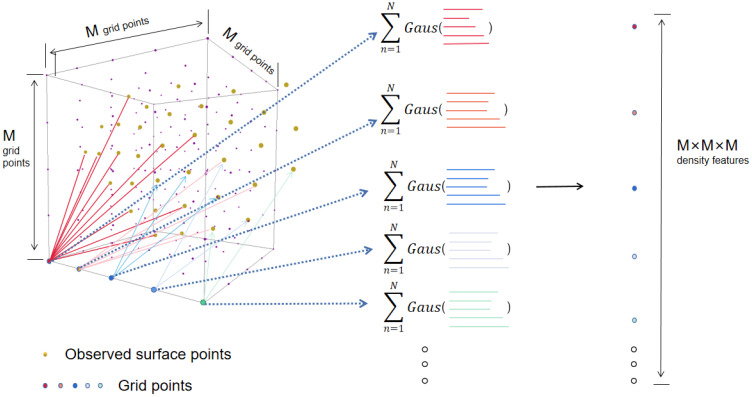
The density feature on each grid point is computed by summing up all the contributions of *N* observed points within this local region (denoted in gold); the overall representation for this region is fixed in size as $M^{3}$, and is invariant to order permutation or the length of input entries.

With the above property, each lattice descriptor abstracts the shape variations with respect to diffeomorphism, while global isometry only influences the result to a limited degree; see Figure 3 for a visualization. Note that the local descriptor can easily be substituted by multiple species of descriptors; for instance, the 3D HoG descriptor [34] defines a process including an explicit conversion from raw point cloud data back to a depth map, then computing the statistics feature on a fixed angled plane to form the representation. However, this kind of operation inevitably loses the fineness of raw scan results, as the depth map yields a regular 2D domain. The curvature descriptors and SIFT feature descriptors rely on transforming 3D point clouds into surface representations with reduced depth information. In contrast, our descriptor captures the density feature within a solid-structured base space, where variations in each axis can be reserved.

### 3.2. Manifold Scattering Transform on Face Point Clouds

**Figure 4:**
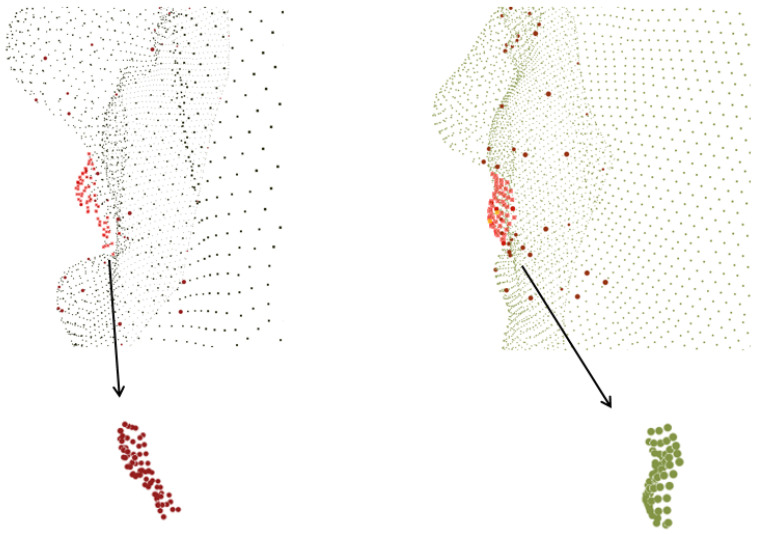
The left-hand image shows a part near the mouth of a sample from 3D-BUFE with an “Anger” expression; the red points are the neighborhood cluster origin at one of the landmarks. The right-hand image shows the same cluster from the same person with a “Happy” expression; a compound deformation can be observed within which the local rotation should not be simply ignored.

With the above local spatial features in hand, we can build a global representation with spectral embedding. Other than embedding all the points into a whole graph or manifold, for 3D FER there is a prior property that can help reduce the computation complexity. First, the description of local geometry is likely to be affected by global as well as local rotations, and applying a rotation-invariant descriptor (as in [35]) or a harmonic descriptor eliminates isometry (as in most spectral embedding methods) leads to the loss of too much information. In the example from 3D-BUFE shown in Figure 4, a neighborhood cluster is found and denoted as points in red; to reserve the finest possible geometric features, our local generic function $\rho_{\chi }$ can be aligned to an underlying surface defined by spectral embedding, as expressions behave locally as both planar stretching/perpendicular haunch-up with rigid rotation. This delicate and significant variable can be decoupled from the global rotation brought by pose variation.

In addition, we note that disturbance induced by translating in $R^{3}$ does not affect this intrinsic representation, as the above embedding is intrinsic to the global isometry, which includes translation. The complexity arising from the arbitrary variation brought about by each subject’s inherent shape becomes more intractable in the case that it entangles the representation. With these considerations, we need to construct a general structure that is stable to global isometry and order permutation while being able to apply the extended convolutional-like operations to align the local signal and compare it as the discriminative feature for recognition.

A scattering transform is a hierarchical framework with a geometric group structure obtained by constructing pre-defined dyadic wavelet layers that has been extended to manifold scattering transform with spectral embedding and diffusion maps [27,36]. With respect to 3D FER, the raw point cloud face can be represented as $\{\rho_{i}{\}}_{c}^{C}\subseteq R^{n}$, where *n* indicates the length of local reference grid point. In practice, this is set as 10×10×10, which is in the form of high dimensional representation. A relatively more common approach is to embed such a generic function into $L_{2}\left(M\right)$ and then implement spectral convolution with a defined spectral path, e.g., diffusion maps or a Random Walk scheme [31].

Alternatively, a direct way to consume point cloud data was proposed in [27] by constructing the heat semi-group process characterized by the operation path $\{H^{t}\}$, where the constructed convolution is defined as

(3) $$H^{t}f=\sum_{k\geq 0}e^{-\lambda_{k}t}\hat{f}\left(k\right)\varphi_{k}$$

where *f* is from exterior feature descriptors such as SHOT [20].

However, our task here is neither about classifying exterior signals on fixed manifolds nor just manifolds; rather, it is about classifying hybrid representations with specified underlying geometry. Based on this observation, we abandon the exterior generic function, instead using the local density feature function from 3.2 to obtain the convolved density feature functions as follows:

(4) $$H^{t}\rho =\sum_{k\geq 0}e^{-\lambda_{k}t}\hat{\rho }\left(k\right)\varphi_{k}$$

Because the above differential configuration of $H^{t}$ enables further associating the negative Laplace Beltrami operator −Δ and constrains the initial condition as $H^{0}=\rho$, a heat process operator on manifold $u_{\rho }$ is provided by the heat equation

(5) $$\partial_{t}\left(u_{\rho }\right)=-\Delta_{x}u$$

Note that we do not simply apply the approximating algorithm from Section 3.2 in [27] to approximate the Laplace Beltrami operator; rather, we utilize the landmarks set ${\left\{p_{landmark_{c}}\right\}}_{c=1}^{C}$ to compute the spectral decomposition, which only undertakes the role of the skeleton to align samples into generic coarse underlying geometry while eliminating the influences of extrinsic isometry. Specifically, we denote D and W as the diagonal degree matrix and affinity matrix of the landmarks point set from regular spectral embedding methods, with N being the length of the landmark sequence and ϵ is the estimated width parameter. The discrete approximation −Δ is provided by

(6) $$L^{N,\epsilon }=\frac{1}{\epsilon N_{landmark}}\left(D-W\right)$$

and most importantly, the wavelet transform can be constructed and specifically parameterized as

(7) $$W_{J}:={\left\{W_{j}\rho \right\}}_{j=0}^{J}\cup \left\{A_{J}\rho \right\}$$

where $W_{0}=Id-H^{1}$ and the global low-pass filter is $A_{J}=H^{2^{J}}$. The diffusion time scale *t*, which here indexes the geometric changes along the increasing width of receptive fields and the wavelets to capture multi-scale information within each scale, can be computed by

(8) $$W_{j}=H^{2^{j-1}}-H^{2^{j}}$$

Then, with the defined wavelets, the first-order scattering moments can be computed as follows:

(9) $$S\rho \left[j,q\right]:=\int_{M}{\left|W_{j}\rho \left(x\right)\right|}^{q}dx$$

where 0≤j≤J and 0≤q≤Q indicate the scaling steps and higher order moments, respectively; an absolute nonlinear operation on the coefficients provides the wanted invariant property within this layer.

By iterating the above procedure, the resulting second-order output is

(10) $$S\rho \left[j,j^{\prime }q\right]:=\int_{M}|W_{j^{\prime }}|W_{j}\rho \left(x\right)|{|}^{q}dx$$

Finally, the *q*th zero-order moments are the integration on $L_{q}\left(M\right)$:

(11) $$S\rho \left[q\right]:=\int_{M}{\left|\rho \left(x\right)\right|}^{q}dx$$

and by concatenating these orders of moments as the overall representation of one sample, they can be input into trained classifiers such as SVM or Neural Networks to accomplish expression classification.

## 4. Experimental Results

For a fair comparison, we conducted experiments using the Bosphorus [13] and 3D-BUFE [14] datasets and compared results with typical methods for 3D FER. The proposed network was implemented on PyTorch [37] and trained on an i7-8700K CPU and a single GTX2080TI GPU. The Bosphorus and Bu-3DFE datasets were utilized as the major material for validation of our methods. One full ten-fold cross-evaluation procedure consumed about 7 h on the Bosphorus dataset and 12 h on the BU-3DFE dataset, and the testing procedure consumed 25 s on Bosphorus and 42 s on 3DBU for each procedure.

### 4.1. Dataset Description

(1) The 3D-BUFE(Binghamton University 3D Facial Expression) dataset [14] contains 2500 facial expression models from 100 subjects (56 females and 44 males) with six prototypical expressions: AN (anger), HA (happiness), FE (fear), SA(sadness), SU (surprise), and DI (disgust). The performance evaluation was based on classifying these six expressions.
(2) The Bosphorus dataset [13] contains 4666 scans collected from 105 subjects with six expressions. It contains relatively more exterior variations (including head poses) and occlusions (hands, hair, eyeglasses) in the samples. We followed the protocol in [3,11,15], which utilizes 65 subjects with 7 expressions denoted as AN (anger), HA (happiness), FE (fear), SA (sadness), SU (surprise), DI (disgust), and NE (neutral).

### 4.2. Evaluation Protocol and Metrics

Following the protocol in [3,15], we applied ten-fold cross-validation on both datasets. To excavate convincing results, we divided each dataset into training/validation/testing splits randomly according to their subjects. A Support Vector Machine (SVM) and full connection network were used for training, with 70% subjects and 20% subjects taken as the validation set and the remaining 10% as the testing set. The results obtained by comparing the related methods are presented in Section 4.4.

For both models, we applied a hyper-parameter tuning process, mainly to the scattering network, where *J* indicates the scaling parameter, *Q* indicates the statistical norm parameter and *k* indicates the embedded dimension searched as the primary parameters. For the fully connected network, we applied stochastic gradient descent as the optimizing method. We generally set the learning rate as 0.001, batch size as 32, and weight decay as 0.001 for both datasets. To complete the comparison we used the Adam optimizer, but did not achieve a better result. For SVM, a Radial Kernel Function (RBF) SVM was applied with a parameter grid search scheme with a range of penalty *C* and kernel width γ. Further details about the parameter tuning process are provided in Section 4.3. The overall prediction accuracy was defined as the mean accuracy of ten prediction times. Confusion matrices for both datasets are provided in Section 4.4.

### 4.3. Hyper-Parameter Tuning Process

The typical size of a sample in Bosphorus and 3D-BUFE ranges from 8 k to 50 k; the resolution of the local reference frame should not be too sparse, as in that case the local feature may be too vague for finer deformations. However, increasing this value can lead to greater computation complexity in the rate of $O(n^{3})$; therefore, we assigned 10×10×10 as the general hyper-parameter of the local frame. Other than the frame resolution, major the hyper-parameters can be divided into two kinds, namely, scattering and classifiers. We began the process of hyper-parameter tuning on BOSPHORUS, with the procedural results illustrated in Table 2. The experiments on 3D-BUFE inherited this setting, except for the categories of labels, which were reduced from 7 to 6. The accuracy rate of facial expression recognition during hyper-parameter tuning on the Bosphorus database is shown in Table 2.

### 4.4. Model Evaluation

**Figure 5:**
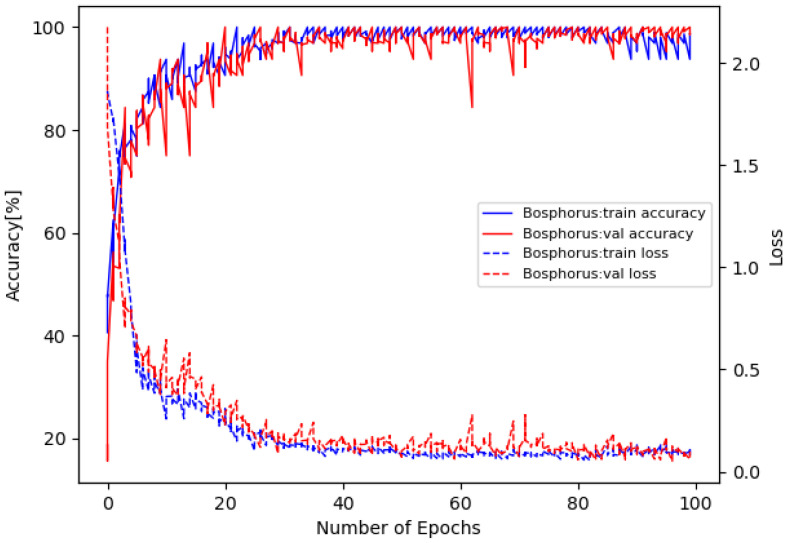
Loss and accuracy on Bosphorus dataset.

**Figure 6:**
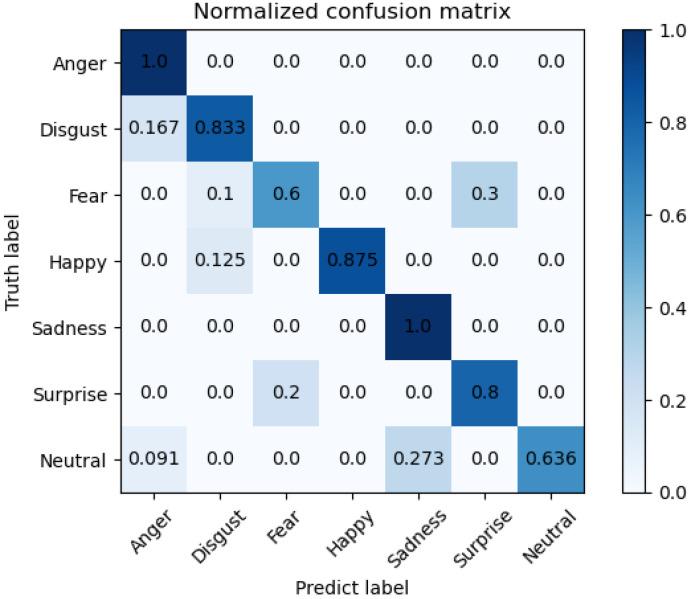
Confusion matrix on Bosphorus dataset for seven expressions.

**Figure 7:**
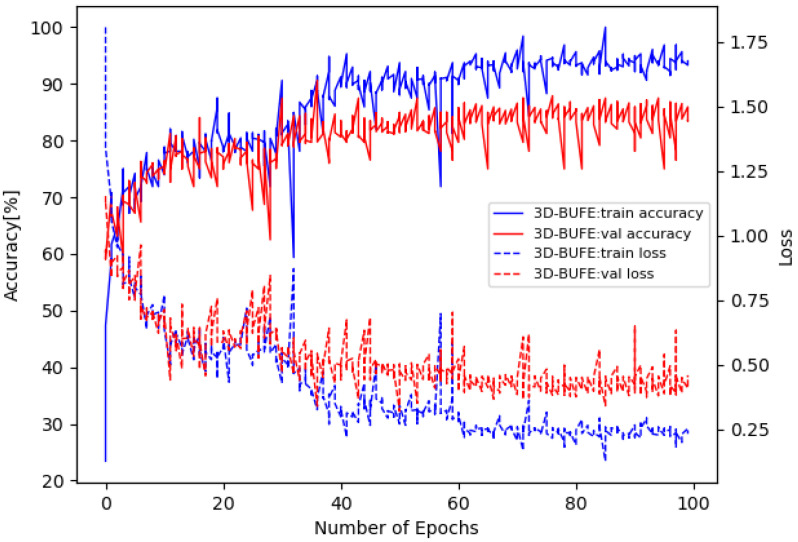
Loss and accuracy on the 3D-BUFE dataset.

**Figure 8:**
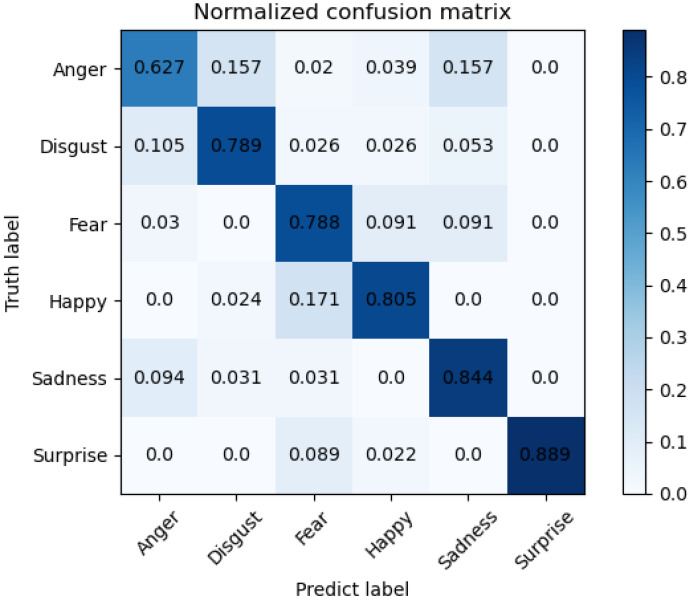
Confusion matrix on 3D-BUFE dataset for six expressions.

The training loss and accuracy curves on Bosphorus are illustrated in Figure 5, and the confusion matrix is in Figure 6. The training loss and accuracy curves on 3D-BUFE are illustrated in Figure 7, and the confusion matrix is in Figure 8. While the training loss decreased to about 0.15 within 30 epochs and then slowly converged at about 0.1 in 50 epochs on Bosphorus, the training loss decreased at a relatively slower pace at the beginning period on 3D-BUFE, then eventually converged at around 0.25. The accuracy with both datasets continued to grow within 40 epochs, then began to swing, and finally converged within a small district. For validation, the shape of the loss curve on Bosphorus has a better outlook, although the results with the testing set indicate a difference in terms of vision. This may be related to the scale difference between these two datasets, with more realizations in 3D-BUFE effectively reducing overfitting during the training procedure.

The overall testing accuracy was 77.55% on the Bosphorus dataset; the best results were achieved with pure point cloud coordination information, which led to an improvement over the other techniques of around 8%. The results of the comparison are stated in Table 3 along with the modalities used in each method.

The confusion matrix on the Bosphorus dataset is shown in Figure 6; we compared individual expression recognition accuracy with others methods, and the superior performance of our model can be seen on Anger (AN) and Sadness (SA). However, there is room for improvement on Neutral (NE) and Surprise (SU). Note that the relatively lowest accuracy on Neutral (NE) might be reasonably considered as a side proof for our theory regarding the fact that the ’averaged shape’ of all the other expressions may be around this point. Furthermore, by looking into the samples from Bosphorus dataset one by one, we noticed that the Fear (FE) and Surprise (SU) samples appear very similar, with mouths and eyes both wide open; only small-scale variations can be observed through deliberate observation, i.e., Surprise (SU) has a slightly more exaggerated degree. This may be related to the limited amount of observations and additional constraining methods, e.g., the attention mechanism may help with balancing.

The comparison with recent methods on the Bosphorus dataset is shown in Table 3 and Table 4. It can be seen that our method has improved performance when recognizing Anger (AN) and Sadness (SA), while it has relatively poor performance on Fear (FE) and Neutral (NE). With respect to the overall accuracy, our method achieves competitive performance with all the other compared methods. It is notable that the use of 3D FER methods on raw point cloud data and on data from other high-resolution sensors continues to progress; our method shows that a more direct approach is possible.

The overall testing accuracy was 78.33% on the 3D-BUFE dataset, which is the best result with pure point cloud coordination on 3D-BUFE. The results of the comparison are stated in Table 5 along with the modalities used in each method. Note that beause the resolution of 3D-BUFE samples is lower, around 8k–10k, the good results provide intuitive evidence for the validity of our model on sparsely scanned samples.

The confusion matrix on the 3D-BUFE dataset is shown in Figure 8. We compared individual expression recognition accuracy with other methods; acceptable performance of our model can be seen on Sadness (SA) and Surprise (SU). The accuracy on Anger (AN) has relatively weaker performance, with about 16% samples misrecognized as Disgust (DI) and another 16% as Sadness (SA).

The comparison with recent methods on the 3D-BUFE dataset is shown in Table 5 and Table 6; it can be seen that our method has comparable performance with SA and SU, as well as with the other mixed modal methods. As discussed above, the size of 3D-BUFE samples limits resolution ability, which may have led to the performance gap with other exterior feature methods; however, it can be seen from the overall accuracy that the method can be further developed.

### 4.5. Stability against Perturbation of Landmark Positions

One interesting effect that may be related is that the choice of manual landmark notation may lead to differences in performance. Because our approach relies on a coarse underlying spectral representation as the entry used to parameterize subsequent scattering networks, certain perturbations in landmark positions should bring about a marginal effect on recognition results. In order to clarify the numerical difference between an accurate landmark and a situation with detection error, we accomplished a controlled experiment with additive white noise being added to the original manual landmark coordinates (x,y,z) to imitate a noisy situation. Specifically, the variance of the noise distribution was set to 10% of the averaged mutual Euclidean distances of each set of landmark points. As a result, we see a minor digression in overall accuracy (see Table 7), which nonetheless surpasses the current best result with GDL in [11].

## 5. Conclusions

In this article, we have presented a geometric deep learning framework with the aim of improving the recognition of 3D point cloud facial expressions with inherent and localized geometric features. By creatively using a manifold scattering transform to construct the general manifold as the coarse structure of an expressed face sample, our work succeeds in capturing discriminative features from local pure point coordination signals, and outperforms the current state-of-the-art competing approaches with PointNet structures. We hope that our approach can inspire the research community to further propel research into achieving greater Facial Expression Recognition capability with high-resolution sensed data and the corresponding representation methods. The proposed solution for 3D FER utilizing GDL methods to represent complex data in this paper indicates wider expansion possibilities. We intend to expand this research to problems that share similar issues or aspects, e.g., identity recognition and micro-expression recognition problems. In addition, more challenging environmental conditions may be thoroughly dealt with for better real-time application.

## Figures and Tables

**Table 1:** Representative methods related to 3D FER emerged in recent years; a trend can be seen in terms of using raw data to learn representations for recognition.

No.	Models	Year	Modality	Classifiers
1	HoG + HoS [21]	2011	3D mesh	SVM
2	Zernike Moments [25]	2011	Depth image	SVM
3	HoG + Differential Mean Curvature Maps [22]	2013	Depth image	SVM
4	SURF + Conformal mapping [3]	2015	3D + 2D	SVM
5	CNN [4]	2016	3D + 2D	NN
6	SPD [15]	2017	3D mesh	SVM
7	RNN [26]	2020	3D + 2D	NN
8	ML-UDSGPLVM [2]	2021	Multi-view 2D image	NN
9	3DMM [17]	2021	3D point cloud	-
10	PointNet [11]	2021	3D point cloud	NN

**Table 2:** Recognition accuracy during the hyper-parameter tuning process.

Hyper-Parameters		Accuracy
*J*	0	77.55%
	2	74.46%
	4	74.16%
	6	72.34%
	8	65.59%
*Q* (Norm)	[0.5, 1]	74.46%
	[0.5, 1, 2]	77.55%
	[0.5, 1, 2, 3]	72.3%
*k*	6	77.55%
	10	70.28%
type of eigenvalues	smallest magnitude (SM)	77.55%
	smallest magnitude (LM)	73.75%
Optimizer	Adam	75.41%
	Momentum	77.55%
Classifier	SVC	74.46%
	ISTC [33] + Full connection network	77.55%

**Table 3:** Comparison of classification accuracy with different methods on the Bosphorus dataset, the best results in each expression are emphasized with boldface.

Method	AN	DI	FE	HA	SA	SU	NE
Vretos et al. (2011)	0.708	0.585	0.431	0.923	0.508	0.477	-
Wang et al. (2013)	0.635	0.706	0.628	0.925	0.745	**0.956**	-
Azazi et al. (2015)	0.825	0.900	**0.863**	0.975	0.675	0.838	0.813
Hariri et al. (2017)	0.863	0.853	0.810	0.930	0.798	0.905	**0.875**
Nguyen et al. (2020)	0.700	0.619	0.573	0.930	0.486	0.775	0.748
Li et al. (2021)	0.870	**0.897**	0.835	**0.998**	0.898	0.917	-
Nguyen et al. (2021)	0.700	0.619	0.573	0.930	0.486	0.775	0.748
Ours	**1.000**	0.833	0.600	0.875	**1.000**	0.800	0.636

**Table 4:** Protocol comparison with state-of-art methods on the Bosphorus dataset.

Method	Accuracy	Classifier/Feature Extractor	Modality	Recognized Expressions
Vretos et al. (2011)	0.605	SVM	3D + 2D	7
Wang et al. (2013)	0.766	SVM	3D mesh	6
Azazi et al. (2015)	0.841	SVM	3D + 2D	7
Hariri et al. (2017)	0.862	SVM	3D mesh	7
Li et al. (2021)	0.903	Transformer	3D + 2D	6
Nguyen et al. (2021)	0.690	PointNet++	3D point cloud	7
Ours	0.783	MST + NN	3D point cloud	7

**Table 5:** Comparison of classification accuracy with other methods on the 3D-BUFE dataset, the best results in each expression are emphasized with boldface.

Method	AN	DI	FE	HA	SA	SU
Berretti et al. (2010)	0.817	0.736	0.636	0.869	0.646	0.948
Azazi et al. (2015)	0.787	0.901	0.737	0.935	0.837	0.945
Huynh et al. (2016)	**0.913**	**0.952**	0.867	**1.000**	0.875	0.957
Hariri et al. (2017)	0.880	0.947	**0.917**	0.978	0.853	0.983
Li et al. (2021)	0.871	0.885	0.863	0.973	**0.877**	**0.980**
Ours	0.627	0.789	0.788	0.805	0.844	0.889

**Table 6:** Protocol comparison with state-of-art methods on the 3D-BUFE dataset.

Method	Accuracy	Classifier/Feature Extractor	Modality	Recognized Expressions
Berretti et al. (2010)	0.775	SVM	3D mesh	6
Azazi et al. (2015)	0.790	SVM	3D + 2D	6
Huynh et al. (2016)	0.927	CNN	3D + 2D	6
Hariri et al. (2017)	0.862	SVM	3D mesh	6
Li et al. (2021)	0.908	Transformer	3D + 2D	6
Ours	0.776	MST + NN	3D point cloud	6

**Table 7:** Comparison of results with noiseless and noisy settings.

Noise Cond.	Datasets	Accuracy	Drop
Noiseless	Bosphorus	78.33%	-
	3D-BUFE	77.55%	-
Noisy	Bosphorus	73.15%	5.18%
	3D-BUFE	75.42%	2.13%

## Data Availability

Not applicable.

## Abbreviations

The following abbreviations are used in this manuscript:

MDPI	Multidisciplinary Digital Publishing Institute
DOAJ	Directory of open access journals
TLA	Three letters acronym
LD	Linear dichroism
